# Genomic Investigations of Spoken and Written Language Abilities: A Guide to Advances in Approaches, Technologies, and Discovery

**DOI:** 10.1044/2025_JSLHR-25-00152

**Published:** 2025-10-29

**Authors:** Simon E. Fisher

**Affiliations:** a Language and Genetics Department, Max Planck Institute for Psycholinguistics, Nijmegen, the Netherlands; b Donders Institute for Brain, Cognition, and Behaviour, Radboud University, Nijmegen, the Netherlands

## Abstract

**Purpose:**

The aim of this tutorial is to show how the rise of molecular technologies and analytical methods in human genetics yields exciting new ways to understand the biological foundations of spoken and written language. The focus is on complementary strategies capturing genetic variation of different kinds (rare gene disruptions and common DNA polymorphisms), discussing how these can be associated with developmental speech, language, and reading disorders as well as with interindividual differences in the general population.

**Results:**

The first half of the tutorial discusses rare variants that are sufficient themselves to cause a severe speech and/or language disorder. This begins with lessons learned from studying *FOXP2* disruptions that cause childhood apraxia of speech, accompanied by impaired language production and comprehension, before considering how genome or exome sequencing has uncovered pathogenic variants in an array of additional neurodevelopmental genes, including *CHD3*, *DDX3X*, *KAT6A*, *SETBP1*, *SETD1A*, *WDR5*, and *ZFHX4*. The second half of the tutorial covers the study of common DNA variants with individually tiny effect sizes, highlighting the challenges of robustly associating them with variability in language-related skills. Against this background, a shift from small candidate gene studies to large-scale genome-wide association study designs is transforming the landscape of the field, gaining leverage from team science approaches and personal genomics. The shift is illustrated with selected examples of recent studies of relevant quantitative measures and diagnostic status in many thousands of participants.

**Conclusions:**

This work demonstrates the dramatic impact genomic innovations are having on the language sciences and how molecular genetic approaches can address long-standing questions about neurobiology and the evolution of distinctive human traits. Potential translational consequences for speech or language pathology vary according to the types of DNA variation and will benefit from enhanced communication about the roles of genomics in clinical contexts.

It has long been known that genetic factors make significant and substantial contributions to our capacities for acquiring proficient spoken and written language (e.g., Pennington & Smith, 1983). Over the past 25 years, scientists have begun to successfully zoom in on the genomic underpinnings of these crucial human traits, using interindividual variation as an entry point (Deriziotis & Fisher, 2017). Such work has been able to benefit from broader technological innovations in DNA collection and analyses (Boycott et al., 2013; Visscher et al., 2017), as well as enhanced statistical methodologies and computational infrastructures (Abdellaoui & Verweij, 2021; Grotzinger et al., 2019). This tutorial describes how these advances have together allowed researchers to identify and study molecular genetic variants that contribute to variations in people's speech, language, and reading skills and explains why this is valuable for understanding human biology. The main body of the tutorial is divided into two parts, covering major complementary areas of the field. The first part focuses on rare gene disruptions with large consequences for molecular functions, unpacking their roles in relevant neurodevelopmental disorders. The second part gives an overview of common genetic variation involving frequent DNA polymorphisms with tiny individual effects, clarifying how they can be effectively used to gain reliable insights into pathways involved in spoken and written language. The tutorial ends with a brief look toward translational potential and by highlighting the main take-home messages from considering this vibrant field of research.

## Discovery and Characterization of Rare Gene Variants

### A Historical Perspective: Studies of the KE Family

Considerable evidence has emerged showing that rare gene disruptions of various kinds can cause developmental speech and language disorders (den Hoed & Fisher, 2020). These are changes at a single genomic locus that, by having a big impact on the functionality of a particular gene or genes, have a large effect on the development of individuals who carry them. Such work benefits greatly from recent advances in DNA sequencing technology (Boycott et al., 2013), but the founding discovery in the literature hails from more than 2 decades ago (Lai et al., 2001), several years prior to the completion of the Human Genome Project (International Human Genome Sequencing Consortium, 2004). This discovery stemmed from investigations of an unusual British family living in London in which a large number of relatives, across three successive generations, had similar difficulties with mastering spoken language (Hurst et al., 1990). The KE family, as it is referred to in the literature, initially drew special attention from researchers not just due to the large numbers of affected members but also because the inheritance pattern of the disorder seemed consistent with that of an autosomal dominant monogenic (single-gene) trait (Hurst et al., 1990). In other words, the way it was transmitted from one generation to the next, affecting around half of the children and with males and females in similar proportions, suggested that the disorder may involve the disruption of just one copy of a single gene and that this gene was not on one of the sex chromosomes. That some cases of developmental speech and language disorders might potentially be explained by monogenic effects was surprising, because the prevailing view in the field was that the etiology of such traits would be complex and multifactorial, involving joint contributions of risk factors across multiple genes.

Due to the intriguing inheritance pattern observed, beginning even before any analyses of their DNA had taken place, the KE family became the focus of intensive study by neuropsychologists, speech pathologists, and linguists, along with debate over the exact nature of the disorder and which impairments are most salient (Vargha-Khadem et al., 1995). A particularly prominent feature, one that some consider to be the core deficit of the disorder, is difficulty in mastering sequences of complex coordinated orofacial movements underlying speech; this was referred to as developmental orofacial/verbal dyspraxia in early papers about the family (e.g., Vargha-Khadem et al., 1998) but is nowadays typically called childhood apraxia of speech (CAS; Morgan & Webster, 2018). Individuals with CAS make inconsistent speech errors that become worse with increased complexity and length of the planned utterance, which are thought to reflect issues with speech motor programming (Morgan & Webster, 2018). Although arising in childhood, the CAS of the KE family persisted throughout life, as was readily apparent from tests of word and (especially) nonword repetition in the mature adults. In the affected individuals of the KE family, the speech problems did not occur alone but were accompanied by variable deficits in a broad array of language-related skills, across vocal and written modalities, extending to receptive abilities and including impairments in grammar comprehension and production (Watkins et al., 2002). Although it has been noted that some members of the family performed poorly on nonverbal cognitive tests, this did not directly segregate with the CAS diagnosis; a number of affected individuals had nonverbal IQ scores that were in the normal range, regardless of their severe speech impairment. Analyses of profiles across different cognitive tests showed that the affected KE family members had more pervasive impairments in the verbal than in the nonverbal domain (Watkins et al., 2002). In summary, although this is not a selective disorder in which one specific aspect of speech or language (or cognition) is disturbed in isolation, the profile is also not consistent with the broad intellectual disability syndromes that have more typically been studied in the genetics literature (Vissers et al., 2016).

Analyses of DNA markers in the affected and unaffected members of the KE family in the late 1990s supported the hypothesis that a single genetic locus could explain the inheritance of the disorder and pointed to a particular region on chromosome 7 as the most likely genomic location (Fisher et al., 1998). Because this predated the first complete draft sequence of the human genome, the researchers went on to build a detailed map of this part of the chromosome, characterizing known and novel genes within the interval, and searched for mutations in those genes in the affected individuals of the KE family (Lai et al., 2000). An important clue came from identification of an independent case of a child (referred to as CS) who was unrelated to the KE family but had a similar phenotypic profile—severe speech apraxia accompanied by expressive and receptive language impairments (Lai et al., 2000). In the CS case, the child, whose parents were both unaffected, carried a de novo (i.e., newly arising) chromosomal rearrangement known as a balanced translocation, in which part of one of his copies of chromosome 7 was exchanged with part of chromosome 5, and the location of the breakage on chromosome 7 occurred in the same genomic interval that had been mapped in the KE family (Lai et al., 2000). Further investigation by the research team revealed that the chromosome 7 breakpoint in the CS case directly disrupted a novel gene (i.e., one that had not yet been identified in the ongoing human genome sequencing efforts) and that this very same gene was mutated, albeit in a different way, in the affected KE family members (Lai et al., 2001).

Although the gene that the researchers had pinpointed was newly discovered, they could use its DNA sequence and the predicted amino acid sequence of the encoded protein to gain important insights (Lai et al., 2001). In particular, toward the far end of the gene, there was a stretch of sequence encoding a well-defined protein domain with known functional properties, called a forkhead box (FOX). Proteins that contain FOX domains are important regulators of gene expression, that is, how much of a gene product is made in a tissue or cell at a given time. The particular structure formed by that specialized domain allows these proteins to bind directly to DNA, enabling them to modulate (increase or decrease) the transcription of target genes (Golson & Kaestner, 2016). Transcription is the process by which DNA sequences of genes are converted into messenger RNA molecules, which are then used as templates for building proteins. Changes in the levels of transcription of a gene thus change the amount of the encoded protein within a tissue or cell. Genes whose role is to regulate the transcription of other genes are known as transcription factors. Genes that encode FOX domains belong to a group of transcription factors that evolved from a single ancestral gene originating in unicellular eukaryotes (Hannenhalli & Kaestner, 2009). FOX genes are classified into subgroups, labeled with different letters of the alphabet, based on comparing the amino acid sequences of their respective FOX domains, which range from 80 to 100 residues in length (Golson & Kaestner, 2016). The gene found to be disrupted in the CS case and the KE family was given the official name *FOXP2*, because it was the second gene to be discovered in the *FOXP* subgroup (Lai et al., 2001). The “*P*” designation indicates that this was the 16th subgroup of FOX transcription factors to be identified in the literature, with “P” being the 16th letter of the alphabet. Based on current information from genome sequences, there are at least 44 different *FOX* genes found in both humans and mice, classified into 19 subgroups from “A” to “S” (Golson & Kaestner, 2016). As transcription factors are active in diverse tissues of the body, these genes act to regulate a wide array of key biological processes in development and throughout life, and they have been implicated in a host of different human diseases (Golson & Kaestner, 2016).

Like other genes that are located on an autosome (i.e., not on one of the sex chromosomes), individuals typically carry two copies of the *FOXP2* gene—one inherited from the person's father and one coming from the mother. In the CS case, the breakpoint of his de novo translocation had directly disrupted one copy of *FOXP2*, leaving him with only a single functioning copy of the gene (located on the intact copy of chromosome 7). The KE family was found to carry a different type of mutation in *FOXP2*, that is, a single nucleotide change, from a guanine (G) to an adenine (A), in the part of the gene that codes for the DNA-binding FOX domain of the encoded protein (Lai et al., 2001). This variant was in a heterozygous state (i.e., affecting one copy of the gene) in all of the 15 affected members of the family across three generations but was not found in any of the unaffected relatives or in a panel of healthy, unrelated control individuals (Lai et al., 2001). Although a small change at the genomic level, the mutation yields a big effect on the encoded protein, substituting a histidine for an arginine at a key evolutionarily conserved point of the FOX domain, in a way that is predicted to prevent it from binding to its usual preferred target DNA sequence. (When a change leads to the substitution of one amino acid for another in the encoded protein, geneticists usually refer to this as a “missense” variant.) On the basis of the two complementary lines of evidence from the CS case and the KE family, Lai et al. (2001) proposed that two functioning copies of *FOXP2* are needed for children to acquire proficient spoken language and that damage to one copy is sufficient to cause a severe speech and language disorder.

### Insights From Investigating *FOXP2*

In the years that followed, further support for the role of *FOXP2* in speech and language development has emerged from a wide array of different sources, including identification of additional independent cases of *FOXP2*-related disorders (MacDermot et al., 2005; Morison et al., 2023; Reuter et al., 2017) and experimental validation of the functional impacts of *FOXP2* protein-coding variants in cell-based systems (Estruch et al., 2016; Vernes et al., 2006). Cellular assays have been developed for assessing multiple properties of the FOXP2 protein, including intracellular localization, DNA sequence binding, regulation of downstream targets, and capacities for protein–protein interaction, and these can be used to systematically test for the potential pathogenicity of DNA variants identified in cases of speech and language disorders (Estruch et al., 2016). For example, cell-based investigations of a mutant protein carrying the arginine-to-histidine substitution in the KE family found that, like normal FOXP2 protein, it is usually located in the nucleus of cells but that it shows a tendency to sometimes mislocalize to the cytoplasm (Vernes et al., 2006). More crucially, this mutant protein also has reduced affinity for the DNA sequences that the FOXP2 protein should normally bind to and is disturbed in its regulation of downstream target genes (Vernes et al., 2006), while still retaining the capacity to interact directly with unmutated FOXP2 protein molecules (Estruch et al., 2016).

Following the discovery of the effects of *FOXP2*, some commentaries and coverage in the popular press mischaracterized it as a/the “gene for speech” or “gene for language.” Although, by now, there is considerable evidence that rare disruptions of *FOXP2* yield a monogenic form of developmental speech and language disorder, such findings do not make it a “gene for speech” (see Marcus & Fisher, 2003), and it is essential to avoid this kind of oversimplistic framing when discussing genetic contributions to human behavior and cognition (Fisher, 2006). As noted earlier, in terms of molecular functions, *FOXP2* encodes a transcription factor, regulating the expression (i.e., increasing or decreasing activity) of sets of downstream target genes (Vernes et al., 2007). A transcription factor does not act alone in a cell but acts through complex dynamic interactions with other regulatory proteins (Estruch et al., 2018). Moreover, depending on which type of cell it is active in and the particular array of interacting cofactors that are also expressed in that cell type, the same transcription factor can play multiple roles in diverse tissues and at different points of development of an organism (Ahmed et al., 2024; French et al., 2019; Shu et al., 2007; Spaeth et al., 2015). In this context, it is important to recognize that although *FOXP2* is expressed in a number of interesting cell types in developing and adult central nervous systems (Ferland et al., 2003; Lai et al., 2003), it is not specific to the brain but also expressed in other sites of the body, including cellular subpopulations of the lung, gut, heart, and pancreas, among other tissues (Schroeder & Myers, 2008).

Another reason for the skepticism over framing *FOXP2* as a mythical “gene for speech” is that, although humans seem to be the only organisms with capacities for spoken language, this gene (like the vast majority of genes of the human genome) is clearly not unique to our species (Fisher & Marcus, 2006). On the contrary, versions of *FOXP2* have been identified and studied in a wide array of diverse vertebrates, across mammals, birds, reptiles, amphibians, and fish (e.g., Bessho et al., 2023; Kato et al., 2014; Lai et al., 2003; Ludington et al., 2024; Lüffe et al., 2021; Mendoza et al., 2015), with the gene showing generally high degrees of evolutionary conservation on the phylogenetic tree. Thus, the mechanistic involvement of *FOXP2* in the speech and language abilities of humans is likely to be built on much deeper evolutionary foundations (Fisher & Marcus, 2006; Scharff & Petri, 2011). This idea has received support from investigations of its roles in brain development and function in nonhuman animal models, including gene manipulation studies of various kinds in mice and songbirds (Fisher & Scharff, 2009). Many insights have emerged from such work, showing, for example, that the activity of this transcription factor in subsets of neurons in the cortex, striatum, thalamus, and/or cerebellum contributes to motor learning and vocalization behaviors, among other skills (e.g., French et al., 2019; Groszer et al., 2008; Norton et al., 2019; Xiao et al., 2021).

In the decades since *FOXP2* was first discovered, as well as accumulating information regarding the complex roles of the gene in the development and function of neural circuits (see den Hoed et al., 2021, for a comprehensive overview), we have learned more about the range of phenotypic consequences of *FOXP2* disruptions in humans, with the rise of next-generation sequencing driving increased identification of potential pathogenic variants (e.g., Reuter et al., 2017). Still, cases and families tend to be scarce, even in genome screens of CAS cohorts (further discussed below; Hildebrand et al., 2020; Kaspi et al., 2023), meaning that the knowledge base on *FOXP2*-related disorders is more limited than that on many other monogenic neurodevelopmental syndromes. The most comprehensive analyses thus far brought together information on 28 individuals, from 17 different families, carrying an array of different pathogenic variants of *FOXP2* (Morison et al., 2023). The variants included five missense variants (i.e., involving amino acid substitutions in the protein) and 12 loss-of-function variants (i.e., leading to an absent, a truncated, or an otherwise nonfunctional protein). In their analyses of these 17 different families (which did not include the KE family because that had already been extensively described), Morison et al. (2023) found that speech disorders were especially prevalent, observed in 92% of cases, including 88% who could be given a diagnosis of CAS. Language impairments, which ranged from mild to severe in impact, were present in 84%, against a background of general cognition that ranged from average performance to mild intellectual disability. Interestingly, seven individuals with relevant test data available had a profile involving a severe language disorder but average nonverbal cognition (Morison et al., 2023), in line with prior findings from the KE family showing that *FOXP2* variants can have disproportionate effects on speech and language development while nonverbal skills are preserved (Watkins et al., 2002). Generally, motor skills were an area of relative strength compared to other abilities; fine and/or gross motor problems were noted in around half of the cases, but diagnoses of developmental coordination disorder or hypotonia were rare in the cohort, and there were no cases of ataxia. The most prevalent comorbidities were sleep disturbance (42%), depression (22%), and anxiety (19%).

In all cases studied by Morison et al. (2023), the variants were present in a heterozygous state (only affecting one copy of the gene). Indeed, there does not appear to be any report in the literature of a person carrying homozygous variants disrupting both copies of *FOXP2*. The absence of identified human cases with homozygous pathogenic variants is not surprising, given that homozygous mutations or knockouts of the gene have been shown to be lethal in mice, causing very severe developmental delays and early death in the first few weeks after birth (Groszer et al., 2008). In other words, a complete loss of *FOXP2* throughout the body appears to be incompatible with life. An interesting point from investigations of humans carrying heterozygous *FOXP2* disruptions is the general lack of symptoms beyond those affecting brain and behavior (Lai et al., 2001; MacDermot et al., 2005; Morison et al., 2023; Reuter et al., 2017), even though the gene is known to be expressed in specific cell types in several other organs and tissues (Schroeder & Myers, 2008). Such selectivity of symptoms, also noted for mice with heterozygous disruptions of this gene, suggests that (at least some) cells in the nervous system are especially sensitive to levels of this transcription factor, whereas nonneural tissues can tolerate a half dosage (i.e., 50% of the normal amount) of functional FOXP2 protein without adverse consequences (Marcus & Fisher, 2003). This observation—that reduced amounts of functional FOXP2 protein are problematic for only a subset of tissues in which the transcription factor is expressed—is well documented for a number of other FOX proteins involved in development and diseases (Golson & Kaestner, 2016).

### Expanding Horizons to Other *FOXP* Genes

As noted earlier, *FOXP2* belongs to a specific subgroup of the FOX transcription factors. Of the three other genes in this subgroup, two (*FOXP1* and *FOXP4*) are especially similar to *FOXP2* and known to be active in developing and adult central nervous systems, among other sites in the body (Ferland et al., 2003; Shu et al., 2007; Spaeth et al., 2015; Teufel et al., 2003). By contrast, *FOXP3* encodes a crucial molecule for regulatory T-cell function, implicated in an immune-related disorder (immune dysregulation, polyendocrinopathy, enteropathy, X-linked syndrome; Bacchetta et al., 2018). A distinctive feature of the “P” subgroup is that the encoded proteins interact with each other to form so-called “dimers,” where two molecules bind to jointly regulate downstream target genes (B. Wang et al., 2003). These might be homodimers, involving the same FOXP protein binding to itself, or heterodimers, involving a mixture of two different FOXP proteins (e.g., FOXP2 bound to FOXP1 or to FOXP4), potentially modulating which downstream targets are regulated (Sin et al., 2015). Moreover, the expression sites of the different neural *FOXP* genes include some regions and cell types in the brain where only one type of *FOXP* is active as well as cells in which two or even all three *FOXP* genes are simultaneously co-expressed (e.g., in neuronal subpopulations in the striatum; Mendoza et al., 2015).

Based on its high sequence similarity to *FOXP2*, the capacity for the encoded proteins to form heterodimers, and evidence of their co-expression in some parts of the brain (Bacon & Rappold, 2012), the *FOXP1* gene has been considered a candidate for being involved in speech and language disorders. Targeted sequencing of *FOXP1* in a cohort of 49 children with a primary diagnosis of CAS did not find any pathogenic variants (Vernes et al., 2009). However, in 2010, Hamdan et al., in their work on cohorts of nonsyndromic intellectual disability and autism spectrum disorder, uncovered the first cases of rare pathogenic variants of *FOXP1*, reporting that the affected individuals showed severe language impairments as part of their phenotypic profile. As with the earlier work on *FOXP2* disruptions, researchers identified different heterozygous missense and loss-of-function variants in affected individuals and applied a range of cell-based assays to demonstrate pathogenicity at the molecular level (Sollis et al., 2016). Intriguingly, several independent cases have been identified to be carrying a *FOXP1* mutation that directly matches the *FOXP2* mutation originally found in the KE family; that is, it is found in a heterozygous state and yields the identical arginine-to-histidine substitution at the same point of the forkhead domain (Lai et al., 2001; Sollis et al., 2017). When cellular models were used to assess functional impact, the arginine-to-histidine substitution had highly similar consequences for transcription factor properties, regardless of which FOXP protein it occurred in, but the associated clinical profiles were notably broader and more severe for the *FOXP1* mutation cases (Sollis et al., 2017). A recent study of parent-reported symptoms in 40 individuals with heterozygous pathogenic *FOXP1* variants found that all cases had developmental difficulties (cognitive, communication, social–emotional, and motor delays), and broader clinical issues in many included bladder control issues, sleep difficulties, eye problems (strabismus, hypermetropia), and abnormal muscle tone (Koene et al., 2024). In-depth investigations of the communication abilities of 29 individuals with pathogenic *FOXP1* variants found atypical speech in all cases; eight were minimally verbal, and all others had dysarthric and apraxic features, mostly accompanied by phonological deficits (Braden et al., 2021). Language scores were generally low, but cases with loss-of-function variants had expressive skills that were preserved as compared to their comprehension abilities (Braden et al., 2021), contradicting prior suggestions that *FOXP1*-related disorders involve relative strengths in receptive versus expressive language (Le Fevre et al., 2013; Meerschaut et al., 2017).

In 2021, *FOXP4* became the third of the *FOXP* transcription factor genes to be implicated in a monogenic neurodevelopmental syndrome (Snijders Blok et al., 2021). The researchers characterized clinical features observed in six unrelated individuals with heterozygous disruptions of *FOXP4*; five carried de novo missense variants affecting the FOX domain of the encoded protein, whereas the sixth had a pathogenic protein-truncating variant. The most consistent phenotypic feature across these cases was delayed development of speech or language skills, especially with regard to expressive abilities; all six individuals had received speech therapy, and two had been given formal diagnoses of expressive language disorder. Despite such developmental delays, half of the cases went on to have normal speech involving full and complex sentences, indicating that this syndrome is milder compared to the disorders associated with pathogenic variants in either *FOXP1* or *FOXP2* (cf. Braden et al., 2021, and Morison et al., 2023, respectively). Most of the *FOXP4* disorder cases had either mild or no intellectual disability, whereas other partly overlapping features across the affected individuals included growth abnormalities, congenital cervical vertebral anomalies, diaphragmatic hernia, and ptosis (Snijders Blok et al., 2021). Just as for *FOXP2* and *FOXP1* before, cellular models were used to assess functional impacts of the identified *FOXP4* variants, providing independent confirmation of pathogenicity. For example, in assays of the respective mutant proteins, all of the FOX domain missense variants identified in the affected individuals showed loss of transcriptional repression activity, and most also displayed disturbances in subcellular localization (Snijders Blok et al., 2021). At this point, the numbers of known cases carrying pathogenic variants are only small, but it can be anticipated that additional affected individuals will be identified over the coming years, leading to further insights into the phenotypic profiles of the syndrome, along with a more extensive comparison to the disorders associated with disruptions of the other *FOXP* genes.

#### Beyond FOXPs: Rare Variant Analyses Implicate Multiple Novel Genes

Only a minority of cases of CAS carry pathogenic variants in *FOXP2*, but considerable progress is now being made in identifying other molecular genetic causes. For example, by now, it is well established that certain types of copy number variants (CNVs), in which stretches of chromosomes spanning multiple genes are either duplicated or deleted, substantially increase the risk of developmental speech disorders, especially CAS. One of the CNVs most clearly implicated in CAS is the 16p11.2 deletion, following the initial discovery of independent recurrent cases reported over a decade ago (Newbury et al., 2013; Raca et al., 2013). This discovery opened up the potential for genotype-first in-depth investigations of the profile of speech and language deficits in carriers (Fedorenko et al., 2016). For example, one notable study gathered a cohort of 55 individuals (47 children, eight adults) carrying 16p11.2 deletions and performed a battery of standardized tests of oromotor skills, speech, language, and general cognition; 77% of the children and 50% of the adults were given a formal diagnosis of CAS, whereas 70%–73% of the children had expressive and/or receptive language impairment (Mei et al., 2018). Multiple independent reports have provided additional evidence of links between 16p11.2 CNVs and speech and language deficits, even after controlling for factors such as a diagnosis of autism spectrum disorder or broader cognitive impairments (Kim et al., 2020; Verbesselt et al., 2024). Recent family-based analyses further confirmed the involvement of 16p11.2 and other CNVs in CAS and speech sound disorders (Chan et al., 2024), offering support for previous proposals that some cases might be explained by the co-occurrence of more than one CNV in the same individual (in line with the so-called “two-hit hypothesis”; Newbury et al., 2013).

In addition to clarifying contributions of CNVs, research of recent years has fostered a more thorough understanding of rare genetic contributions to CAS, driven by the availability of methods for rapid, cost-effective DNA sequencing of entire genomes or exomes (the exome is the subset of the genome that encompasses protein-coding genes). The first whole-genome sequencing study of a speech disorder investigated 19 unrelated individuals with a formal diagnosis of CAS and for whom pathogenic *FOXP2* variants were already excluded (Eising et al., 2019). For nearly half of the cohort, genome sequencing could also be done for the proband's father and mother, both of whom were unaffected; by comparing the genomes, it was possible to systematically search for potential de novo variants that might be responsible for the disorder. Indeed, in a third of such cases, convincing pathogenic de novo variants were found disrupting protein-coding genes involved in brain development, implicating the *CHD3*, *SETD1A*, and *WDR5* genes (Eising et al., 2019).

Although no recurrent mutations were identified in this initial, modest-sized cohort, for each locus, follow-up studies further clarified the etiological relevance of the gene, providing valuable new insights into the severity and range of phenotypes associated with pathogenic variants. For example, on the basis of the initial index case from the CAS cohort, Snijders Blok et al. (2018) used a genotype-driven approach to assemble a collection of 35 independently ascertained individuals with de novo pathogenic variants of *CHD3*, most of which were missense mutations clustering in the ATPase/helicase domain of the encoded protein, with the potential to disturb the chromatin modeling functions of that protein. The encoded protein is known to belong to a complex (assembly) of proteins that are important for regulating early brain development, based on studies in mice (Goodman & Bonni, 2019), but little is known at present about why disruptions lead to issues with speech development in humans. Consistent with the profile of the index case, the researchers found that speech delay or disorder was present for all assessed individuals (Snijders Blok et al., 2018). On the other hand, for general intellectual functioning, a broad profile was seen with considerable variability in severity across different probands, even when they carried similar or identical pathogenic variants. Cognitive abilities ranged from borderline to severe intellectual disability, with an even distribution of individuals in the intermediate levels in between. Additional features associated with this newly defined monogenic disorder included hypotonia, macrocephaly, facial dysmorphisms, joint laxity, and vision problems, all of which showed variability in presence and/or severity from one proband to another. Further work confirmed this variability in the expressivity of symptoms and uncovered a role for not only de novo but also inherited variants, even those that are transmitted from an unaffected or a mildly affected parent (van der Spek et al., 2022).

Similar stories could be told for the CAS-associated *SETD1A* and *WDR5* genes (Eising et al., 2019). *SETD1A* encodes a histone methyltransferase that modifies histone molecules at the promoters (upstream regulatory regions) of active genes (Kummeling et al., 2021). (Histones are proteins that are crucial for DNA packaging in eukaryotic cells, and so, modifications of histones play key roles in the regulation of gene expression.) Intriguingly, the protein encoded by *WDR5* also participates in protein complexes that affect gene regulation via modification of histones. Moreover, like *CHD3*, as well as being linked to CAS, both *SETD1A* and *WDR5* have also been implicated in broader neurodevelopmental syndromes that go beyond speech symptoms (Kummeling et al., 2021; Snijders Blok et al., 2022). These examples highlight a more general point for studies of rare variation in speech disorders. They show how de novo confirmed pathogenic variants identified in probands with isolated CAS (i.e., without comorbid disorders or intellectual disability) might represent cases at the extremes for a monogenic neurodevelopmental syndrome with a spectrum of variable phenotypic symptoms (Snijders Blok et al., 2018; van der Spek et al., 2022). Such observations are arising more and more as we expand our understanding of the contributions of rare gene disruptions to speech and language disorders; at the molecular level, the boundaries between different types of neurodevelopmental syndromes are much more blurred than we might have originally anticipated.

In the original Eising et al. (2019) CAS genome sequencing study, considering all genes identified with either de novo or rare pathogenic variants, the researchers found that multiple were linked to the regulation of neurodevelopment and that most belonged to a shared module of genes that are co-expressed together during the early ontogeny of the human brain. As noted above, the study was limited by the modest number of cases available for sequencing, reducing the likelihood of identifying independent recurrent mutations within the same screening cohort. However, during the past 5 years, multiple additional larger cohorts of unrelated probands with CAS have been screened using whole-genome or whole-exome sequencing, with sample sizes of 34–70 individuals (Hildebrand et al., 2020, *N* = 34; Kaspi et al., 2023, *N* = 70; Formicola et al., 2024, *N* = 34). As well as confirming the high burden of rare genetic disruptions contributing to CAS, with 21%–32% of the probands in these studies carrying high-confidence pathogenic DNA variants, together, the investigations so far point to several genetic loci that are recurrently mutated across cohorts, including *DDX3X*, *KAT6A*, *SETBP1*, *SETD1A*, and *ZFHX4* (Eising et al., 2019; Formicola et al., 2024; Hildebrand et al., 2020; Kaspi et al., 2023). Thus, in-depth analyses of speech and language in the relevant monogenic syndromes have become especially pertinent (e.g., Forbes et al., 2024; Morgan et al., 2021; St John et al., 2022). Interestingly, pathogenic variants of *SETD1A* not only have been found in CAS cohorts (Eising et al., 2019; Kaspi et al., 2023) but also were separately uncovered in genome sequencing studies of idiopathic speech delay (Eising et al., 2024), representing a case of a gene implicated across multiple different speech-related disorders. Functional follow-up is underway for several of the genes identified through the CAS screens using a range of human cell culture models, including 2D and 3D neuronal cultures derived from induced pluripotent stem cells, with the aim of understanding how different rare variants yield similar or different profiles in the people who carry them (den Hoed et al., 2024; Wong et al., 2025).

## Studying Common Genetic Variation

As a complement to investigations of the roles of rare genetic variants of large effect to a phenotype of interest, another major branch of human genomics examines the contributions of common polymorphisms, usually defined as variations in DNA sequence that occur at an allele frequency of at least 1% in the general population (Visscher et al., 2017). Modern human populations harbor millions of common polymorphisms distributed throughout the genome, and, by now, these have been extensively mapped and cataloged (International HapMap 3 Consortium, 2010). Crucially, much of the polymorphic content of the human genome has little impact on gene regulation or function, involving DNA variants that have become common simply by spreading neutrally (i.e., without any consequence for distal traits, hence imparting no selective advantage or disadvantage). Nonetheless, within the background noise of benign variation, there exist some common polymorphisms that do have functional impact, ultimately making contributions to interindividual variability in anthropometric, clinical, and behavioral traits as well as acting as risk factors in disorders (Visscher et al., 2017). As a consequence of advances in complex trait genetics, we now know that the effect size of any one polymorphism by itself is typically tiny and that the combination of a great many different common variants in aggregate may explain a substantial proportion of the variation in the trait or disorder of interest. To give an example from one of the most well-studied phenotypes in human genetics, very large-scale studies of height have identified a set of more than 12,000 polymorphic sites in the genome that together account for up to 40% of the interindividual variability in this trait in samples of European ancestry (Yengo et al., 2022).

Most early efforts to find associations between common DNA variants and aspects of behavior/cognition were based on unrealistic expectations about the simplicity of genetic underpinnings and the likely effect sizes of the polymorphisms being investigated (assuming that these effect sizes would be much larger than what we now know to be biologically plausible). Initial work in this area was constrained by the fact that only a few polymorphic markers were known at the time, from just a tiny handful of genetic loci (see Border et al., 2019, for examples of the most prominent). Researchers again and again focused their attention on a small number of candidate genes and one or two common polymorphisms known to map within them, usually chosen because of the biological pathways they were linked to, leaving the potential contributions of the majority of loci and variation in our genomes uncharted and unexplored (Border et al., 2019). The limited genomic scope of such endeavors was compounded by limitations in scale, with studies examining gene–trait associations in small samples (tens of participants) that lacked power to robustly detect realistic effect sizes of common polymorphisms, often without adjusting for the multiple-testing burden of exploring different ways of classifying phenotypes or grouping different genotypes (see Grabitz et al., 2018). An accompanying problem is that these kinds of underpowered study designs also suffer from elevated false-positive rates (Button et al., 2013). Although such studies were prolific for quite a while in the literature, many of the conclusions from this kind of “candidate gene” work have now been called into question by systematic, well-powered studies of key polymorphisms in much larger samples of many thousands of participants (e.g., Border et al., 2019). (Note that these criticisms apply to studies of *common DNA variants* in particular candidate genes, in contrast to the work done on *rare DNA variants of known large effect*, described in prior sections of this tutorial, which usually does not suffer from such limitations.) Further discussion of these issues as they apply to genetic investigations of speech and language, including the important distinctions between effects of rare and common variants, can be found in Uddén et al. (2019).

Contemporary reevaluation of findings from the candidate gene era has been largely driven by a combination of advances in molecular technologies and statistical methods over the past 2 decades (Abdellaoui & Verweij, 2021; Visscher et al., 2017). In particular, with the advent of DNA microarrays in the early 2000s, it became possible to carry out low-cost parallel genotyping of millions of single-nucleotide polymorphisms (SNPs) located at different sites spread throughout the genome, enabling researchers to comprehensively capture common genetic variation in a way that could be scaled up to ever-larger samples. This technical development fueled the rise of the genome-wide association study (GWAS), a research design in which successive SNPs located at different positions across all chromosomes are tested individually to see whether their allelic status is associated with a trait of interest (McCarthy et al., 2008). The approach can be (and has been) flexibly applied to a very wide range of target phenotypes of different kinds. For example, this could be a qualitative diagnosis of a disorder, for which allele frequencies are compared between cases and matching controls, or a quantitative measure (e.g., height, body mass index, blood pressure), with researchers testing for a relationship between the allelic status and magnitude of that measure (Visscher et al., 2017). Regardless of the nature of the trait, given that a standard GWAS involves systematic mass univariate screening with millions of different SNPs, the statistical thresholds for identifying significant associations should always take into account the considerable multiple-testing burden, to avoid false-positive findings (McCarthy et al., 2008). Considering the tiny effect sizes expected for individual SNP associations and the rigorous thresholds associated with genome-wide testing, a GWAS depends on large cohorts in order to ensure sufficient power for a reliable characterization of the underlying genetic contributions, involving samples of tens of thousands, even hundreds of thousands, of participants (Visscher et al., 2017). In fact, some studies of phenotypes that are particularly easy to measure have, by now, assembled information across millions of individuals; for example, the study of height by Yengo et al. (2022) brought together GWAS data from roughly 5.4 million people of diverse ancestries.

### Challenges for Investigating Language-Related Traits

How have these dramatic shifts in complex trait genetics impacted studies of speech, language, and reading, and what might GWAS approaches reveal about the underlying biology? Although early work on gene mapping in reading disability (for example) was at the forefront of the field (see Fisher & DeFries, 2002, for a review), genetic investigations of language-related traits then lost momentum and came to lag some way behind those of biomedical phenotypes. Writing in 2017, Deriziotis and Fisher highlighted several factors that had contributed to this comparative lack of progress. One of the biggest impediments at that time was a lack of suitably large cohorts of individuals with matched data from molecular genetics and phenotypes with relevance to spoken and written language abilities. Large-scale GWAS efforts often leverage the fact that standard batteries of clinical and/or neuropsychological tests are routinely deployed in medical and psychiatric contexts, meaning that measures of similar phenotypes of interest may be opportunistically available across multiple cohorts for meta-analysis. This is one of the reasons why the largest GWASs published thus far in the field of complex trait genetics have been for commonly collected anthropometric traits such as height and body mass index (e.g., Yengo et al., 2022). Unfortunately, speech, language, and reading traits are seldom included in standard biomedical screens, such that most currently available clinical/psychiatric cohorts are not informative for genetic studies in the language sciences (Deriziotis & Fisher, 2017). Moreover, even now, these traits are typically ignored in major high-impact biobanking initiatives, such as the UK Biobank, which has collected in-depth phenotypic and high-resolution molecular genetic data from half a million adults across Great Britain (40–69 years old at recruitment; Sudlow et al., 2015).

Additional factors highlighted by Deriziotis and Fisher (2017) concern the complexity of defining the target trait in a way that is feasible for connecting to data at the genetic level. Our language capacities involve many different facets that can be potentially characterized and studied in human participants: expressive and receptive skills of diverse kinds related to lexicon, phonology, (morpho) syntax, semantics, pragmatics, and beyond. Moreover, these are moving targets known to change through the lifespan, especially early in development and in late adulthood, and we still have much to learn about the nature of inter- and intraindividual variations in phenotypic profiles. Finally, there is an obvious extra complication for researchers interested in gathering data from measures of language skills at scale, one that does not arise for most other traits studied in contemporary genetics/genomics research, in that languages can themselves differ in different countries of the world.

### Enhancing the Power of Genome Screens With Team Science Approaches

The above-noted challenges have significantly slowed down progress, as compared to the broader field of complex traits. Some studies have been able to gain initial insights into genetic architecture by applying GWAS approaches to family-based cohorts or population isolates (Benchek et al., 2021; Kornilov et al., 2016). Moreover, in the past 2–3 years, well-powered, large-scale, genome-wide investigations of speech, language, and reading skills have finally become feasible (e.g., Doust et al., 2022; Eising et al., 2022; Verhoef et al., 2024), in part through the adoption of team science approaches similar to those that proved effective in tackling other genetically complex human traits. One prominent example is the founding of GenLang, an international consortium bringing together scientists from multiple different countries across the world who are interested in researching the genetic underpinnings of traits related to spoken and written language, not only for disorders but also for interindividual variation in the normal range (https://www.genlang.org/). The consortium was established with three primary stated goals: (a) to develop a network of scientists who actively engage in sharing ideas and expertise in relation to genetics of spoken and written language; (b) to provide a flexible dynamic platform for carrying out high-powered, genome-wide screening of speech, language, reading, and related skills via meta-analyses of existing data sets; and (c) to find ways of harmonizing approaches to phenotype definition and collection across different cohorts, including developing and optimizing web-based testing tools. At the time of writing, the GenLang consortium includes almost 100 researchers from 18 countries spread across the world.

The potential of the team science approach is illustrated by one of the first outputs from the GenLang consortium, which provided a genome-wide perspective on individual differences in an array of quantitatively assessed reading- and language-related skills (Eising et al., 2022). The study was based on integrating already available phenotype and genotype data in 22 cohorts from 12 different countries (United Kingdom, France, Austria, Germany, Netherlands, Switzerland, Spain, Hungary, Finland, United States, Canada, and Australia), focusing pragmatically on traits for which the largest sample sizes could be achieved. Much of the work to support an initiative of this kind involves aligning the genotype and (especially) phenotype data to make a meta-analytic approach feasible; for example, cohorts may have ascertained their target populations in different ways, collected participants across different age ranges, or used different versions of psychometric tests even when tapping into similar underlying constructs. Because this was an international study, seven different languages were represented, although the majority of participants (in cohorts from the United Kingdom, United States, Canada, and Australia) primarily spoke English. Based on these cross-cohort phenotype alignment efforts, there were five quantitatively assessed traits that would yield reasonable combined sample sizes for pursuing a GWAS strategy: word reading (19 cohorts; *N* = 33,959), nonword reading (13 cohorts; *N* = 17,984), spelling (15 cohorts; *N* = 18,514), phoneme awareness (12 cohorts; *N* = 13,633), and nonword repetition (10 cohorts; *N* = 14,046). Even with total sample sizes of this magnitude, it was only the largest GWAS, for word reading, that pinpointed an individual genomic locus exceeding the genome-wide significance threshold. Three genes were mapped in the vicinity of this association signal, namely, *DOCK7*, *ANGPTL3*, and *USP1*, encoding a guanine nucleotide exchange factor involved in neurogenesis, a growth factor specific for the vascular endothelium, and a deubiquitinating enzyme involved in Fanconi anemia, respectively.

Crucially, even without pinpointing individual, genome-wide significant, association signals, the data from a suitably informative GWAS can provide valuable insights for understanding the trait of interest, using newly emerged methods for molecular genetic epidemiology (Grotzinger et al., 2019). For each of the five reading- or language-related measures in their GenLang study, Eising et al. (2022) went on to assess SNP heritability, a measure of how much of the trait variance can be accounted for in aggregate by common DNA variation across the genome. These SNP heritabilities were all significant and ranged from 13% for nonword repetition to almost 26% for nonword reading. Aggregate signals of this magnitude are in line with those for other complex human behavioral/cognitive phenotypes (Abdellaoui & Verweij, 2021) and allow for further follow-ups addressing diverse questions such as whether there are genetically mediated overlaps with other traits (which can be assessed in other samples) or if there are disproportionate contributions from genomic regions of particular evolutionary interest (Alagöz et al., 2022). When Eising et al. (2022) applied genomic structural equation modeling (Grotzinger et al., 2019) to the GWAS data, the model showed that a shared genetic factor could account for the majority of cross-trait variation in word reading, nonword reading, spelling, and phoneme awareness and that this shared factor had only a partial overlap with the genetic underpinnings of general cognition, as indexed by measures of performance IQ and reports on levels of educational attainment. Interestingly, nonword repetition stood somewhat apart from the other GenLang traits in this analysis. Further investigations of multivariate genetic signals across word reading, nonword reading, spelling, and phoneme awareness suggested neurobiological links to individual differences in the surface area of the banks of (i.e., the cortical tissue flanking) the left superior temporal sulcus, as assessed independently in the UK Biobank (Eising et al., 2022), a part of the brain previously linked to the processing of spoken and written language (Wilson et al., 2018). Among other findings, these multivariate genetic signals were also associated with the regulation of gene expression in fetal human brain tissue (Eising et al., 2022).

One big limitation of the GWAS meta-analysis (meta-GWAS) efforts carried out thus far, a characteristic of the field of human complex trait genetics as a whole, is that they have largely drawn on populations with European ancestry. Recent genomic studies in cohorts from Hong Kong and China are beginning to address this (Lin et al., 2024; Z. Wang et al., 2023), but much work is left to be done to engage broader cohorts from across the world, against diverse population backgrounds and with respect to a wider range of languages.

### Gaining Leverage From Personal Genomics Initiatives

The example of the GenLang quantitative trait meta-GWAS illustrates the importance of being able to scale up investigations to enable robust analyses of genomic architecture. At the same time, the low number of individual genome-wide significant associations shows that, even with sample sizes in the tens of thousands, power remains an issue. Thus, the field needs smart strategies for further scaling up, to achieve samples of hundreds of thousands of individuals, for example, through the development of reliable online test batteries (Hintz et al., 2024) that can be deployed in cohorts with existing molecular genetic information. A complementary strategy that allows very large sample sizes to be ascertained is epitomized by a recent GWAS study of dyslexia reported by Doust et al. (2022). In this investigation, researchers identified 42 independent dyslexia-associated loci that exceeded thresholds for genome-wide significance (Doust et al., 2022), representing a dramatic increase in informativeness compared to prior studies of this kind, even those completed only a year before (Gialluisi et al., 2021). A major contributor to the enhanced power of the 2022 dyslexia GWAS was the very substantial increase in sample size over previous investigations, involving 51,800 independent cases and 1,087,070 control individuals (Doust et al., 2022). This scaling up of over 20-fold, compared to the largest prior dyslexia case–control GWAS, was achieved through a scientific collaboration with the personal genomics company 23andMe, deriving case or control status based solely on a “yes” or “no” answer to the single question, “Have you been diagnosed with dyslexia?” Clearly, there is a trade-off here between the precision and depth of phenotypic characterization and the magnitude of the sample sizes that can be attained.

Crucially, multiple lines of further investigation were used to validate that the simple self-report of dyslexia status was capturing biologically meaningful genetic signals related to deficits in people's reading skills. First, the genetic signals were largely consistent across males and females as well as ages from different points of the lifespan. Second, the researchers could use the GWAS data available from the 23andMe cohort and from the parallel GenLang quantitative trait study (Eising et al., 2022) to assess genetic correlations—a measure of what proportion of genetic influences are shared across two different phenotypes (even those that have been separately assessed in independent cohorts with different participants). The simple self-report of dyslexia status demonstrated 70%–75% genetic sharing with psychometric measures of spelling, word reading, and nonword reading from GenLang and 62% genetic sharing with phoneme awareness, indicating that the yes/no question taps into much of the same underlying genetic architecture as that revealed by directly assessed quantitative tests (Doust et al., 2022; Eising et al., 2022). (Note also that these genetic correlations all had a negative sign, consistent with the notion that a dyslexia diagnosis should be associated with reduced performance on spelling, reading, and phoneme awareness skills.) In contrast, dyslexia status showed much more modest levels of genetic sharing (only 19%) with nonverbal (performance) IQ, demonstrating that self-report of the dyslexia phenotype indexes a trait that is much more closely related to reading and spelling than to general cognition (Doust et al., 2022). Third, the researchers performed another validation step using polygenic indices, a measure of genetic liability to a phenotype, based on the susceptibility alleles that a person carries at many different points across the genome, weighted by the allelic effect sizes estimated from the original GWAS (Abdellaoui & Verweij, 2021; Plomin & von Stumm, 2022). Polygenic indices based on the 23andMe dyslexia GWAS could explain up to 6% of trait variance in reading skills ascertained using quantitative tests in independent cohorts (Doust et al., 2022), which is modest but in the range seen for other complex behavioral/cognitive traits (Abdellaoui & Verweij, 2021; Plomin & von Stumm, 2022). Note, however, that polygenic indices can show clearly significant associations in group-level analyses while still having very limited reliability for predicting a phenotype at the individual level (Wesseldijk et al., 2024).

Of the 42 genetic loci identified in the 23andMe dyslexia GWAS, 15 pointed to genes connected to cognitive ability and/or educational attainment in prior studies, whereas 27 were newly implicated without any prior links to general cognition (Doust et al., 2022). The availability of this large-scale systematic screen for dyslexia-associated loci also gave opportunities for unbiased assessment of the potential impact of prior candidate genes (e.g., as reviewed by Carrion-Castillo et al., 2013). None of the common DNA variants reported in prior small-scale studies of reading/language traits were among the genome-wide significant loci (Doust et al., 2022). Moreover, there was no enrichment of pathways underlying neuronal migration, a prominent account of etiology in earlier genetic studies (Guidi et al., 2018).

As with other large-scale investigations thus far, the dyslexia study was limited by the population background of the GWAS sample, which primarily comprised people with European ancestry. Another limitation was the use of a single self-report question for defining the trait of interest, although the accompanying genetic correlation and polygenic index analyses demonstrated that this question reliably captured biological factors underlying quantitatively measured reading skills. Beyond dyslexia, similar self-report screening approaches have now been extended to other traits such as developmental language disorder, as in this study of more than 46,000 individuals from the Danish Blood Donor Study (Nudel et al., 2023). A complementary design for scaling up GWAS efforts incorporates information from school performance grades across a range of skills related to oral/written language and mathematical abilities, available from genetically informative samples comprising many thousands of people (Rajagopal et al., 2023). Overall, the above GWAS examples demonstrate how we are opening a new chapter in studies of contributions of common DNA variation to speech, language, and reading traits, enabling follow-up studies on genetically mediated connections to neuropsychiatric traits and disorders (Ciulkinyte et al., 2025), biological/evolutionary overlaps with other phenotypes such as musicality (Alagöz et al., 2025), links to interindividual variation in structural and functional neuroimaging phenotypes (Amelink et al., 2024; Soheili-Nezhad et al., 2024), and relationships with spatiotemporal gene expression patterns in the human brain (Wong et al., 2024), among other innovative research lines for the future.

## Clinical Significance and Take-Home Messages

The potential clinical significance of these advances in speech or language genomics is broad and varied, depending on the type of DNA variations involved, and would require a full article for a thorough discussion. A few points of interest are highlighted here, in connection with the distinction between rare and common variations that frame the current tutorial. The biggest prospects for clinical translation are with regard to rare DNA variants. Investigations of severe developmental speech disorders indicate that as many as 32% of children with a formal diagnosis may carry a pathogenic protein-coding variant with a large effect at the molecular level (Eising et al., 2019; Formicola et al., 2024; Hildebrand et al., 2020; Kaspi et al., 2023). This high yield of monogenic causes suggests that integrating genome or exome sequencing into the standard clinical workflows of speech-language pathologists could make a substantial impact, so long as it is coupled with increasing clarity of communication about how we should interpret genetic effects. Much effort is underway toward using knowledge on the relevant genes to develop novel therapeutics targeted to affected individuals (a topic that is beyond the scope of the present tutorial). Such translational work will likely take years before yielding concrete outcomes in the clinic. Nonetheless, even before such targeted therapeutics are realized, we should not underestimate the value of a genetic diagnosis for the cases and families involved, through possibilities of connecting with other cases or families with similar variants, learning more about the profiles of the strengths and weaknesses associated with a particular syndrome, and enhancing understanding of what to expect as the affected children develop through life.

In contrast, when it comes to common DNA variation, the diagnostic value of polygenic indices (encompassing information for variants of small effect across many different genes) remains limited at the present time (Wesseldijk et al., 2024). As illustrated in the earlier sections of this tutorial, such indices are becoming informative for investigating questions of clinical relevance in group-based comparisons, such as the genetically mediated relationships between different types of disorder (Abdellaoui & Verweij, 2021). At the same time, their predictive power at the individual level is still very low. Even after higher powered GWAS efforts have given us more reliable polygenic indices for disposition to language-related conditions, there will remain considerable uncertainty in the relationship between genotypes and phenotypes, meaning that such indices will need to be used in combination with other types of diagnostic markers. One exciting area for the future concerns the integration of information from rare and common variations to help understand how these different types of DNA effects come together to both increase risk and modulate severity/profile across different individuals with the same condition.

In summary, this tutorial has highlighted how complementary strategies that capture DNA variation at different ends of the frequency spectrum are helping to decipher the genetic underpinnings of spoken and written language, for disorders and interindividual differences in the general population. Early work in this area revealed how rare disruptions of genes that regulate brain development can lead to disorders with disproportionate effects on speech and language. The tutorial discussed how, more than 20 years after the discovery of *FOXP2*, genomic advances are pointing to further genes with rare variants disrupting speech to additionally reveal broader links to neurodevelopment, blurring the boundaries across disorders. As argued here and elsewhere (den Hoed & Fisher, 2020; Fisher & Scharff, 2009), studies of the critical genes in humans as well as in cellular and animal models can give powerful molecular windows into the relevant pathways. Moving to individual differences in speech, language, and reading, the tutorial outlined how these are influenced by a great many common DNA polymorphisms of tiny effect, requiring large samples, a rigorous study design, and integrated approaches for robust detection and analysis. Characterizing the relevant phenotypes at scale is crucial for success but has so far proved very challenging for language-related traits. Nonetheless, the selected examples highlighted here show how, after decades of relying on candidate gene approaches, systematic, large-scale genome-wide scanning is transforming the landscape of this ever-expanding field. In turn, such initiatives open novel research avenues for the language sciences and hold promise of new insights into deeper neurobiological and evolutionary questions, along with potential for clinical advances related to diagnostics and treatment.

## Data Availability Statement

Data sharing is not applicable to this tutorial as no data sets were generated or analyzed. The tutorial references data and results presented in previously published works, all of which are cited.

## Notes

**Publisher Note:** This article is part of the Forum: Research Symposium on Genetics in Communication Sciences and Disorders.
